# Iktishaf+: A Big Data Tool with Automatic Labeling for Road Traffic Social Sensing and Event Detection Using Distributed Machine Learning

**DOI:** 10.3390/s21092993

**Published:** 2021-04-24

**Authors:** Ebtesam Alomari, Iyad Katib, Aiiad Albeshri, Tan Yigitcanlar, Rashid Mehmood

**Affiliations:** 1 Faculty of Computing and Information Technology, King Abdulaziz University, Jeddah 21589, Saudi Arabia; EAlomari0011@stu.kau.edu.sa (E.A.); IAKatib@kau.edu.sa (I.K.); aaalbeshri@kau.edu.sa (A.A.); 2 School of Architecture and Built Environment, Queensland University of Technology, 2 George Street, Brisbane 4000, QLD, Australia; tan.yigitcanlar@qut.edu.au; 3 School of Technology, Federal University of Santa Catarina, Campus Universitario, Trindade, Florianópolis 88040-900, SC, Brazil; 4 High Performance Computing Center, King Abdulaziz University, Jeddah 21589, Saudi Arabia

**Keywords:** smart cities, big data, event detection, road traffic, distributed machine learning, automatic labeling, social media, data analytics, social media analytics, Arabic tweets

## Abstract

Digital societies could be characterized by their increasing desire to express themselves and interact with others. This is being realized through digital platforms such as social media that have increasingly become convenient and inexpensive sensors compared to physical sensors in many sectors of smart societies. One such major sector is road transportation, which is the backbone of modern economies and costs globally 1.25 million deaths and 50 million human injuries annually. The cutting-edge on big data-enabled social media analytics for transportation-related studies is limited. This paper brings a range of technologies together to detect road traffic-related events using big data and distributed machine learning. The most specific contribution of this research is an automatic labelling method for machine learning-based traffic-related event detection from Twitter data in the Arabic language. The proposed method has been implemented in a software tool called Iktishaf+ (an Arabic word meaning discovery) that is able to detect traffic events automatically from tweets in the Arabic language using distributed machine learning over Apache Spark. The tool is built using nine components and a range of technologies including Apache Spark, Parquet, and MongoDB. Iktishaf+ uses a light stemmer for the Arabic language developed by us. We also use in this work a location extractor developed by us that allows us to extract and visualize spatio-temporal information about the detected events. The specific data used in this work comprises 33.5 million tweets collected from Saudi Arabia using the Twitter API. Using support vector machines, naïve Bayes, and logistic regression-based classifiers, we are able to detect and validate several real events in Saudi Arabia without prior knowledge, including a fire in Jeddah, rains in Makkah, and an accident in Riyadh. The findings show the effectiveness of Twitter media in detecting important events with no prior knowledge about them.

## 1. Introduction

### 1.1. Smart Cities, Tranportation, and Social Sensing

Smart cities and societies aim to revolutionize our daily lives and improve social, economic, and environmental sustainability through increased technology penetration, participatory governance, and wise use of natural and other resources [1]. Smart urban and rural developments require timely sensing and analysis of diverse data produced by various edge sensors, smart devices, GPS, cameras, and the Internet of Things (IoT) [2]. Social media such as Twitter have become an important class of sensors for smart urban and rural developments [3], and in many sectors of smart cities and societies, it is increasingly being seen as a conveniently available and relatively inexpensive source of information compared to physical sensors [4]. Road transportation that is considered the backbone of modern economies is one such sector. It costs globally 1.25 million deaths and 50 million human injuries annually and therefore it is a research and development area of high significance.

Increased urbanisation is giving rise to the evolution of cities into megacities where traffic congestion is a leading problem causing devastating economic, social, and ecological losses. The annual cost of congestion in the US is USD305 billion, not to mention the damages to health and the number of deaths. Congestion is caused due to the steadily growing traffic in the cities over the years, road damages, roadworks, traffic accidents, bad weather, and other contingencies. There is a need to detect these causes or events to enable timely planning and operations.

Many times, congestion is caused due to events that are beyond the direct scope of physical road sensors, and therefore physical sensors cannot detect these events until the effects of these events are visible on the roads and can be sensed by the on-road sensors. For example, a football match in a city is likely to disrupt the traffic and increase pressure on the road network in certain segments of the city. Such an event can be detected through social media in advance of the event and timely intervention may reduce the aggravation of congestion in the city. Events such as a major football event may have already been known to the authorities. However, social media can also detect events that are being arranged on ad hoc bases—such as social gatherings, small sports gatherings—and, though these are small, there can be many of these in a city and can create an aggregately large pressure on the city roads. Similarly, unpredictable events such as a fire in a city segment may also disrupt the city traffic and such events can also be detected automatically on social media before their effects on the roads are visible. Moreover, historical analysis of social media data can reveal hidden information related to the traffic that may have not known otherwise and can be used for urban planning.

Twitter is one of the most popular microblogging media used for communication and sharing personal status, events, news, etc. [5]. Twitter allows users to post short text messages called tweets. A massive amount of real-time data is posted by millions of users on various topics including transportation and real-time road traffic [4,6,7,8]. In the recent decade, the use of Twitter and other social media by researchers and practitioners to study different issues in many application domains and sectors has steadily increased [9,10,11,12,13,14]. Transportation is no exception where social media has been used to study various aspects such as for analysing travel behaviours [15], recognizing mobility patterns [16], congestion detection [17], and event detection [4,6,9,18,19]. Due to the microblogging and real-time nature of Twitter, people are likely to communicate information about small and large-scale social gatherings, sports events, or events such as a fire, weather, allowing such information to be extracted in real-time [20]. Such information can allow the detection of transportation-related events and their causes for timely planning and operations. However, while manifesting great potential, several major challenges need to be overcome before its wide adoption in transportation and other areas.

### 1.2. Summary of the Proposed Work

The aim of this work is to develop big data technologies for detecting road traffic-related events (i.e., events that may affect road traffic) from Twitter data in the Arabic language with a focus on Saudi Arabia. Over the past few years, we have continued to build a detailed literature review on social media analytics in transportation. We have learnt from the literature review that the cutting-edge on big data-enabled social media analytics for transportation-related studies is limited. Many more studies are needed to improve the breadth and depth of the research on the subject in several aspects to establish maturity in this area. The research gaps relate to the focus of the studies, the size and diversity of the data, the applicability and performance of the machine learning methods, the diversity in terms of the social media languages, the scalability of the computing platforms, and others [13,21]. The maturity of research in this area will allow the development, commercialization, and wide adoption of the tools for transportation planning and operations (for the literature review and research gap, see Section 2).

This paper brings a range of technologies together to detect road traffic-related events using big data and distributed machine learning. The paper contributes to most of the above-mentioned research gaps. The most specific contribution of this research is an automatic labelling method for machine learning-based traffic-related event detection from Twitter data. In principle, the method itself is generic and can be applied for natural language processing (NLP) in any language. However, in this paper, the method is applied to Twitter data in the Arabic language. One of the approaches to detecting events from social media requires text classification using supervised classification algorithms. Supervised classification requires labeling of data for the training phase. For big data, the manual labeling process is time-consuming and labor-intensive [22]. Using the automatic labelling techniques developed in this paper we are able to deal with over an order of magnitude larger dataset compared to our earlier work in [6]. We are able to detect several real events in Saudi Arabia without any prior knowledge, including a fire in Jeddah, rains in Makkah, and an accident in Riyadh. The proposed automatic labeling method uses predefined dictionaries to reduce the effort, time, and cost of manual labeling of tweets. The dictionaries have been generated automatically for each event type using the top vocabularies extracted from the manually labeled dataset. Then, the dictionaries are adjusted manually to add synonyms and make sure that we do not miss any important vocabulary. After that, we divide them into levels based on the importance and the degree of relevance to the event type. Then, we calculate the weight for each labeled tweet (see Section 3 for details of the tool design including the automatic labelling method).

**Figure 1:**
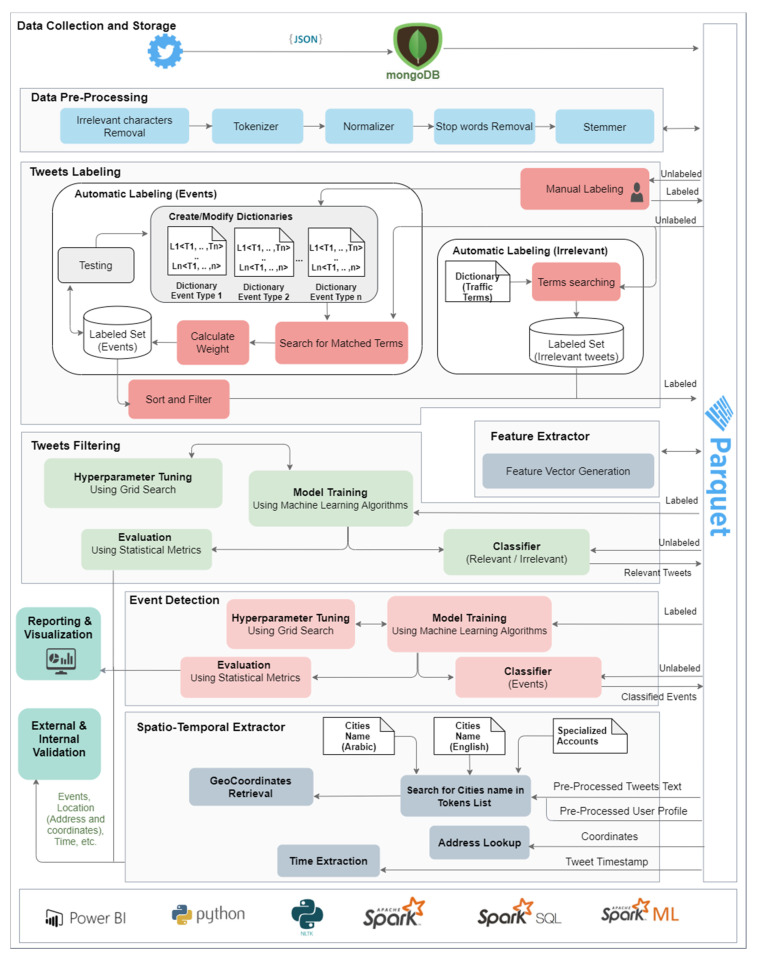
Iktishaf+: The proposed system architecture.

The proposed method has been implemented in a software tool called Iktishaf+ (an Arabic word meaning discovery) that is able to detect traffic events automatically from tweets in the Arabic language using distributed machine learning over Apache Spark. The tool is built using nine components that are used for nine specific functions namely data collection and storage, data pre-processing, tweets labeling, feature extraction, tweets filtering, event detection, spatio-temporal information extraction, reporting and visualization, and internal and external validation. The architectural blocks of the Iktishaf+ system are depicted in Figure 1 (we will describe in detail the system architecture including its nine components in Section 3). Iktishaf+ is built using a range of technologies including Apache Spark, Spark ML, Spark SQL, NLTK, PowerBI, Parquet, and MongoDB.

The Iktishaf+ tool uses Iktishaf Stemmer that we introduced in [6]. It is a light stemmer for the Arabic language developed by us. It is designed to strip affixes based on the length of the tokens. It allows reducing the feature space and minimizing the number of removed letters from the token to prevent changes in meaning or losing important words. We also use in this work a location extractor developed by us that helps to find the location of the detected events. It uses multiple methods to extract the event location. The location is extracted from the tweet text where the place name is explicitly mentioned in the message or is included as hashtags. Additionally, the event locations are identified from the account names using a predefined list of account names that are specialized in posting about traffic conditions in different cities in Saudi Arabia. If no information is found, the other attributes associated with the tweets JSON object such as coordinates and user profiles are checked for the location information. These methods have allowed us to extract and visualize spatio-temporal information about the detected events.

The specific data used in this work comprises 33.5 million tweets collected from Saudi Arabia using the Twitter API for a period of over a year. We have not used this data in any of the earlier works. The findings show the effectiveness of the Twitter media in detecting important events, and other information in time, space, and information-structure with no earlier knowledge about them. The detected events are validated using internal and external sources.

Iktishaf+ is an enhanced version of our tool Iktishaf that was introduced in [6]. The tool Iktishaf+ extends the functionality, capacity, and testing of our earlier work on traffic event detection. The earlier work on event detection has reported analyses of 2.5 million tweets [6]. The number of tweets was limited by our ability to manually label the tweets. We have also applied the Iktishaf tool to detect government measures and public concerns related to CVOID-19 using unsupervised learning [13]. Our earlier work on big data social media analytics has also focused on application areas including public sentiments analysis of government services [23], logistics [24,25], and healthcare [7] in both Arabic and English languages.

The Iktishaf+ tool uses open-source big data distributed computing technologies that enable the scalability and integration of transportation software systems with each other and with other smart city systems such as smart healthcare and urban governance systems. An elaboration of the novelty, contributions, and utilization of this work is given in Section 2.4.

The organization of the paper is as follows: Section 2 highlights the related work, in which we review different techniques for traffic events detection from social media in addition to the existing approaches for labeling large-scale datasets. Section 3 describes the proposed methodology and details the tool design and architecture. Section 4 explains the analysis results, which is followed by conclusions and future work reported in Section 5.

## 2. Literature Review

Digital societies could perhaps be characterized by their increasing desire to express themselves and interact with others, and this is done through various digital platforms [26]. The core ingredients of these digital societies, or digital platforms that enable these societies include a range of emerging technologies and their convergence. The technologies include big data [27,28,29,30], high-performance computing (HPC) [31,32,33,34], artificial intelligence [35,36,37,38], cloud, fog, and edge computing [39,40,41], social sensing [42,43,44,45], and Internet of Things (IoT) [36,40,46,47,48]. The applications include transportation [4,49,50,51,52,53], healthcare [7,54,55,56], and others [57,58,59,60]. The pulse for sensing and engaging with the environments are provided by social media and IoT. Sentiment analysis, or opinion mining, is a vital tool in natural language processing (NLP) [61], and many of the notable works on sentiment analysis rely on artificial intelligence, Twitter, and other social media.

We review here the literature related to the topics of this paper, which is detection of events related to road traffic using Twitter in the Arabic language. We begin with the literature about traffic event detection and then we discuss the solutions for automatic labeling. We discuss in Section 2.1 the works that have been developed for event detection in any language whether they use big data or not. Section 2.2 discusses works in the Arabic language and since works for Arabic are very limited, we introduce the studies related to detect any type of events not only traffic events. In Section 2.3, we present the solutions for labeling large datasets. Finally, Section 2.4 reveals the research gap.

### 2.1. Traffic Events Detection Using Social Data (Any Language)

Sakaki et al. [62] proposed an earthquake reporting system using Japanese tweets. They classified real-time tweets into positive (event-related) and negative (not related to events) classes using an SVM classifier. To prepare the training set, they used three groups of features for each tweet, which are keywords in a tweet, the number of words, and the words before and after the target-event words. Furthermore, they extended their work to extract events from tweets referring to driving information [63]. They collected tweets using a list of keywords about traffic-related events such as heavy traffic, traffic restriction, police checkpoints, parking, and rain mist. As in their previous work, they prepared the features using different methods and then they selected the best features to train a classifier using the SVM algorithm. Moreover, Klaithin and Haruechaiyasak [10] analyzed tweets in the Thai language to extract traffic events. They trained a classifier using Naïve Bayes to classify the tweets into six categories, which are accident, announcement, question, request, sentiment and traffic condition. They applied machine learning classifier based on Naive Bayes Model.

Kumar et al. [64] trained a sentiment classification model to detect negative sentiment about a road hazard from Twitter. The data is collected using search filtering with specific terms that relate to traffic. Then, naïve Bayes, K-nearest-neighbor and the dynamic language model (DLM) are used to build models to classify the tweets into a hazard and not hazard. Semwal et al. [65] applied real-time spatio-temporal analysis on Facebook data to detect traffic insights. They designed a module to detect the occurrence of events based on the spike in the number of posts at a specific time period and location. When the number is more than a threshold, the posts associated with that time and location are analyzed to evaluate their sentiments. Further, a random forest classifier was used to predict the most dominant issue for the next day. To address the problem of having an imbalanced dataset, they used SMOTE. Tejaswin et al. [66] also used random forest classifier to predict traffic incidents. The traffic incidents are clustered and predicted using spatio-temporal data from Twitter. The location information is extracted using NLP and background knowledge by using Freebase API, which is a community-curated structured database containing large number of entities and each one defied by multiple properties and attributes that helps in entity disambiguation.

Moreover, D’Andrea et al. [11] collected real-time Italian tweets and classified them after applying text mining techniques. The tweets are classified into three classes namely, traffic due to an external event, traffic congestion or crash, and non-traffic. They built a set of traffic events detected from official news websites or local newspapers and then they compared the time of detecting an event from these official sites with the time of detection from Twitter’s stream fetched by their system.

None of the above-discussed approaches have used big data technologies. Salas et al. [44] used apache spark to process tweets and train model using SVM classification algorithm to classify them into traffic and non-traffic related tweets. To extract location information, they used a combination of name entity recognition (NER) such as Stanford NER and a knowledge base such as Wikipedia.

Suma et al. [67] built a classification model using logistic regression with stochastic gradient descent to detect events related to road traffic from English tweets using Apache Spark. Lau [42] used the latent Dirichlet allocation (LDA) topic modeling module to filter traffic messages. In addition, they used the Spark MLib library and trained classifiers using SVM, KNN and NB to detect traffic events. A detailed survey of event detection techniques using twitter data can be found in [68].

### 2.2. Traffic Events Detection Using Social Data (Arabic Language)

A very limited number of studies have proposed to analyze Arabic social information for traffic event detection, so first we review the studies about detecting any event not necessarily related to traffic. Then, we review the works that focus on transport and traffic events. Finally, we discuss the works that use big data. Alkouz and Alghbari [69] analyzed English and Arabic data, including standard Arabic and UAE dialectical posts from Twitter and Instagram to detect and predict traffic jams. They filtered the collected data to keep only traffic-related data. They used about 2.4 million tweets and 319,125 traffic-related image captions from Instagram. Further, the text is cleaned, tokenized and then stemmed using the NLTK root stemmer. Then, they used a predefined list of keywords to classify the posts into reporting posts or non-reporting posts where reporting posts contain at least one vocabulary from the list. They developed a tool to identify locations from the text of posts and/or GPS location. Further, they employed a linear regression model to predict future traffic jams. Moreover, Alkhatib et al. [70] analyzed tweets written in Modern Standard Arabic and Dialect Arabic analysis for the purpose of incident and emergency reporting. To detect incidents and disasters occurring in the UAE, they collected tweets in real-time using specific keywords, which are a car accident, earthquake, drought, hailstorm, heatwave, building collapse, riot and civil disorder. They labeled 8000 tweets manually to generate a training set and collected 82,150 tweets as a testing set. They built classification models using five machine learning algorithms, which are Polynomial Networks (PN), NB, KNN, Rachio (RA) and SVM. Moreover, they applied root stemmer and the results showed that it improves the classification accuracy. Further, they built NER corpus using Wikipedia to identify certain types of NEs such as building name, event risk and impact level and number of casualties. To extract location information, they built a dictionary of terms related to location names in the Dubai city.

Other researchers have proposed a solution to detect events but their main focus was not on traffic. AL-Smadi and Qawasmeh [71] extracted events about technology, sports, and politics using unsupervised rule-based technique. Alsaedi and Pete [72] developed a solution using naïve Bayes and online clustering algorithms to detect disruptive events. Alabbas et al. [73] detected a high-risk flood using a SVM classifier.

However, none of the above-discussed approaches for event detection from Arabic have used big data technologies. Alomari and Mehmood [18] developed a dictionary-based approach using SAP HANA, which is an in-memory processing platform to analyze Arabic tweets related to traffic congestion in Jeddah city. Additionally, they extracted traffic congestion causes. Furthermore, they extended their work and applied sentiment analysis on traffic-related tweets [19]. Moreover, they developed a supervised classification models using Apache Spark platform to detect eight types of traffic events, which are accident, roadwork, road closure, road damage, road condition, fire, weather, and social events. The results show that SVM achieves better results compared to logistic regression and Naive Bayes algorithm. Subsequently, they extended their work and validate the ability of the proposed Iktishaf tool [6] in detecting various events, their locations and times, with no earlier knowledge about the events from about 2.5 million tweets. Further, they designed a new light stemmer, Iktishaf Stemmer, for Arabic text and study the effect of using it on the performance. The results show that the performance of the trained model with and without using the proposed stemmer is almost similar. On the other hand, comparing to other light stemmers such as Tashaphyne and ISRI, Iktishaf Stemmer helps to minimize the number of letters removed and eliminate changes on the meaning especially for the words that related to transportation.

### 2.3. Solution for Labeling Large Scale Dataset (Any Language)

Manual labeling is a very challenging and expensive process especially with having a very large dataset and thus supervised learning is hard to applied on big social data. One of the solutions is crowdsourcing by cooperating with freelancers but one of the issues is the quality of the work [74]. Besides, crowdsourcing is not a fully automatic approach. In this section, we discuss the existing works that are similar to us and enables labeling text automatically to eliminate the need for human experts to label all the training set.

Pandey and Natarajan [75] proposed a system to extract situation awareness (SA) information and location from Twitter during disaster events. They suggested using semi-supervised classification instead of the traditional supervised machine learning approach, which would be tedious and time-consuming in term of labeling. For creating a semi-supervised model, they manually labeled a small set of tweets and then fed them to the SVM to classify them into situation awareness and non-situation awareness. Then, they used the result from this initial classification to self-train the model. However, their model achieved very low precision and recall value for situation awareness class.

Shafiabady et al. [76] suggested using an unsupervised clustering approach such as self-organizing maps (SOM) and correlation coefficient (CorrCoef) to group the unlabelled documents and use them as labelled data to train the SVM for text classification. However, their approach was applied on documents not on short text such as tweets. Ghahreman and Dastjerdi [77] applied semi-automatic labelling by combining co-training algorithms with the similarity evaluation measure. They labelled a small set of data manually, then they used the SVM algorithm to classify the unlabelled document. After that, based on the threshold, part of the output is selected. Then, they calculated the similarity between the selected documents and manually labelled documents

Zewen et al. [78] suggested labeling a few documents automatically using the external semantic resources e.g., HowNet. Then, they combined the labeled data and most of the unlabeled training data to train the classifier by semi-supervised learning. To label the documents automatically, they obtained the knowledge of the category name using lexical databases as external semantic resources and then they generated a set of features for the corresponding category. Further, they extracted features from the documents as a corresponding feature vector. After that, the similarity between each category name and each text document are calculated to rank the documents and classify them into the corresponding category. Triguero et al. [79] provided a taxonomy for the self-labeled techniques. One of the techniques is the addition mechanism. It consists of a variety of schemes, including incremental, batch and amending.

### 2.4. Research Gap, Novelty, Contributions, and Utilization

It can be seen from the literature review provided in this section that the works that use big data technology for traffic-related event detection are limited. To the best of our knowledge, none of the existing works for Arabic have used big data technology and platforms. Furthermore, none of them have used automatic labeling to address the problem of manual labeling of large datasets. The cutting-edge on big data-enabled social media analytics for transportation-related studies is limited. Many more studies are needed to improve the breadth and depth of the research on the subject in several aspects to establish maturity in this area. The research gaps relate to the focus of the studies, the size and diversity of the data, the applicability and performance of the machine learning methods, the diversity in terms of the social media languages, the scalability of the computing platforms, and others [13,21]. The maturity of research in this area will allow the development, commercialization, and wide adoption of the tools for transportation planning and operations.

The range of technologies that we have incorporated in the Iktishaf+ tool advances the state-of-the-art on big data social media analytics in the Arabic language in a number of ways (some of these contributions related to big data analysis also apply more broadly to English and other languages). Firstly, the extended tool Iktishaf+ has contributed multiple big data pipelines and architectures for event detection (in transportation and other sectors) from social media using cutting-edge technologies including data-driven distributed machine learning and high-performance computing. Secondly, it incorporates a novel pre-processing pipeline for Saudi dialectical Arabic that includes irrelevant characters removal, tokenizer, normalizer, stop words removal, and an Arabic light stemmer to improve event detection and overall performance. This will help many other works in the Arabic language to benefit from our work. Thirdly, the tool incorporates a range of lexicon-based, supervised, and unsupervised machine learning methods for event detection from social media in the Arabic language to enable smarter transportation and smarter societies. Using these methods, we have detected various physical and conceptual events such as congestion, fire, weather, government measures, and public concerns. Fourthly, the extended tool incorporates an automatic labeling method to reduce the effort, time, and cost of manual labeling of large datasets. We are not aware of any automatic labelling work in the Arabic language. Fifthly, we have developed and incorporated methods in the tool for spatial and temporal information from Twitter data to allow spatio-temporal clustering and visualization of detected events. Sixthly, we have developed methods for validating the detected events using internal and external sources. None of the existing works in English and other languages, particularly Arabic, have reported a similar analysis of Twitter data for event detection in terms of the richness of the methods, depth of analysis, and significance of findings. To the best of our knowledge, no work in the Arabic language exists that has used automatic labelling or big data tools or has reported analysis of a large number of tweets such as we have in this paper.

The scalability of the software systems for big data analytics is critical and is being hampered due to the challenges related to the management, integration, and analysis of big data (the 4V challenges). The use of big data distributed computing technologies is important because it will allow the scalability and integration of transportation software systems with each other and with other smart city systems. The ability of the Iktishaf+ tool to execute in parallel could save a month of computing time for the specific dataset size and the problem addressed in our work and speed up the development process [13]. For larger datasets, executing sequential codes may not even be possible, or distributed computing could save years of development time.

The utilization possibilities of our tool are many. For example, governments could learn about the various events, public concerns, and reactions related to certain government policies, measures, and actions (in pandemic and normal times) and develop policies and measures to address these concerns. The public could raise their concerns and give feedback on government policies. The public could learn about various public and industry activities (such as fires, social events, and other events, and economic activities detected by our tool in the earlier work [13]) and get involved in these to address financial, social, and other difficulties. The standardization and adoption of such tools could lead to real-time surveillance and the detection of transportation-related or other events, or disease outbreaks (and other potentially dangerous phenomena) across the globe and allow governments to take timely actions to prevent various risks, the spread of diseases, and other disasters. The international standardization of such tools could allow governments to learn about the impact of policies of various countries and develop best practices for national and international response.

## 3. Iktishaf+: Methodology and Design

Figure 1 illustrates the Iktishaf+ architecture. It consists of nine components, which are: (1) Data Collection and Storage Component, (2) Data Pre-Processing Component, (3) Tweets Labeling Component, (4) Feature Extractor Component, (5) Tweet Filtering Component, (6) Event Detection Component, (7) Spatio-Temporal Extractor Component, (8) Reporting and Visualization Component and (9) External and Internal Validation Component. The next subsection explains the tools and libraries used to develop Iktishaf+. Section 3.2, Section 3.3, Section 3.4, Section 3.5, Section 3.6, Section 3.7, Section 3.8, Section 3.9 and Section 3.10 elaborate each component in detail.

### 3.1. Tools and Libraries

Iktishaf+ is built over the Apache Spark platform, which enables in-memory processing on distributed data. The main libraries that have been used are Spark ML and Spark SQL. Spark.ML is a new package introduced in Spark 1.2. Unlike the Spark.MLlib package that was built on top of RDDs, Spark.ML contains higher-level API built on top of DataFrames creating and tuning practical machine learning pipelines. Moreover, the script was written using Python and runs on the Aziz supercomputer, a Fujitsu 230 TFLOPS machine comprising around 500 nodes, each with 24 cores. Besides, Fujitsu Exabyte File System (FEFS) has been used to provides high performance storage space as well as Scalable I/O performance. FEFS is a scalable parallel file system based on Lustre. The Aziz supercomputer supports running Spark with YARN, which allocates resources across applications.

**Figure 2:**
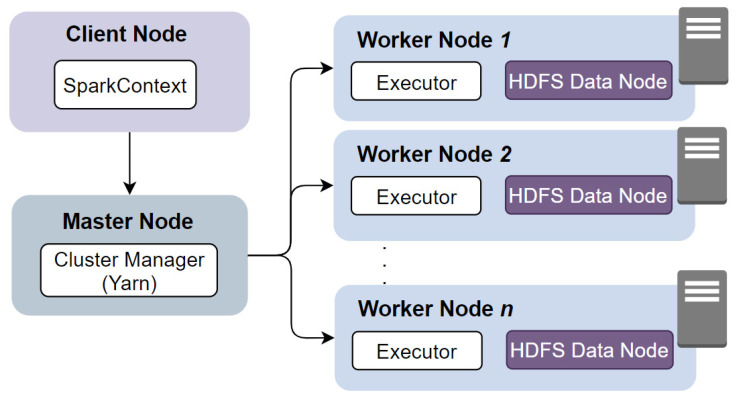
Spark Application Run on Yarn.

Figure 2 shows the architecture of Apache Spark with YARN. Spark applications can run as independent sets of processes on a cluster. It acquires *Executors* on cluster nodes. The SparkContext is responsible for coordinating the application and enable connecting YARN. Then, it sends *tasks* to the executors to run computations for the application.

### 3.2. Data Collection and Storage Component (DCSC)

**Figure 3:**
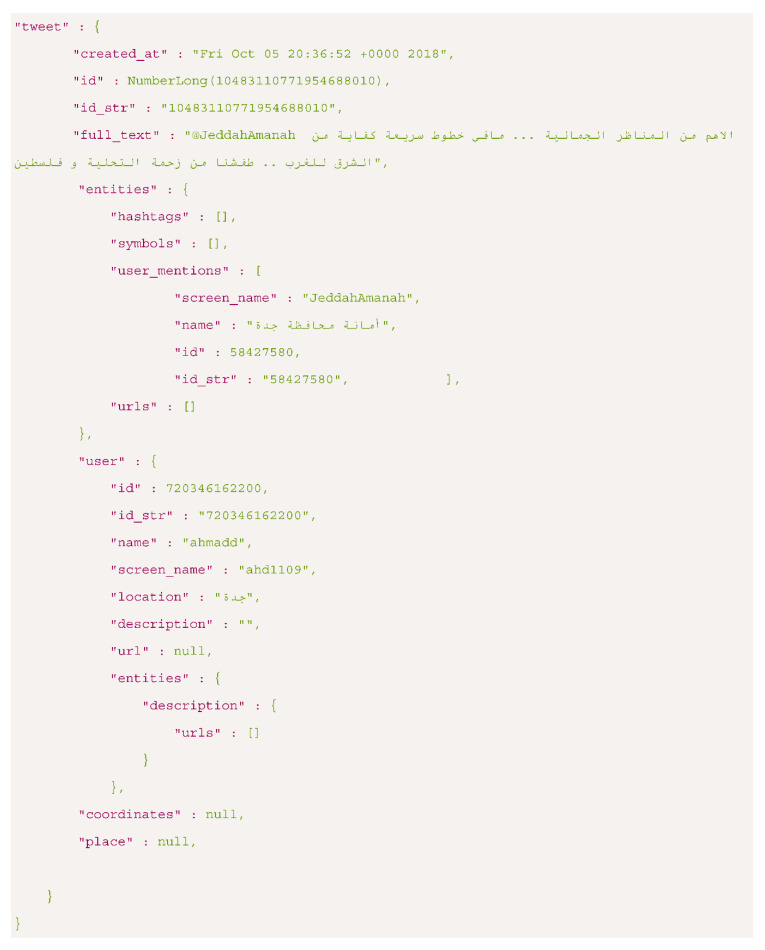
A Tweet Object.

We collected the data using the Twitter REST API, which enables collecting historical data. The returned data by the Twitter API are encoded using JavaScript Object Notation (JSON). Each tweet object includes a unique ID, the text content itself, a timestamp that represents when it was posted, and many child objects such as ‘user’ and ‘place’. Based on their documentation [80], a tweet can have over 150 attributes associated with it. Each child object encapsulates attributes to describe it. For instance, the ‘user’ object contains ‘name’, ‘screen_ name’, ‘id’, ‘followers_count’, and others. Some of the attributes belong to ‘user’ object can be filled in manually by the user such as ‘description’ and ‘location’. Additionally, each tweet includes the ‘entities‘ object, which encapsulates several attributes such as ‘hashtags’, ‘user_mentions’, ‘media’, and ‘links’. The example in Figure 3 shows the core attributes of a tweet object.

We fetch Arabic tweets posted by the users in Saudi Arabia by using geo-filtering. Besides, we searched for tweets using hashtags that included cities name. Both methods ensure collecting tweets about Saudi Arabia or posted from any place inside it, but not necessarily related to road traffic. Moreover, we created a list of specialized accounts that post about transportation and traffic condition in Saudi Arabia and then use them to obtain traffic-related tweets. We collected data in the period between September 2018 and October 2019. The total number of the collected tweets is 33.5 million. After that, we clean the data by removing duplicates and the retweets.

For storing the collected tweets, we need a storage method that provides flexible schemas to store/retrieve the data. So, we found that the NoSQL databases are more appropriate comparing to the traditional table structures in relational databases. One of the common types of NoSQL databases is a document-oriented database. In this type, each key is paired with the document of various document data types, such as XML and JSON. One of the most widely used document-oriented databases is MongoDB. Thus, we used MongoDB to store the fetched tweets using Twitter API.

Moreover, we use parquet file storage. The Parquet is a column-oriented format. It is supported by many data processing systems including Apache Spark. Furthermore, it enables very efficient compression. We select it to store the output after each stage because it is efficient and provides good performance for both storage and processing. Besides, Spark SQL supports both reading and writing Parquet files that automatically preserves the schema of the original data. After reading data from the Parquet file, it is stored in Spark DataFrames, which is equivalent to a table in a relational database or a data frame in R/Python. However, it provides richer optimizations.

### 3.3. Data Pre-Processing Component (DPC)

We use pre-processing component proposed in our earlier work, Iktishaf [6]. Algorithm 1 shows the Iktishaf+ pre-processing algorithm. It received the collected tweets, the Arabic diacritics [D], punctuations [P], and Arabic stop words [SW] as an input while the output is clean, normalized, and stemmed tokens. The collected tweets are exported from MongoDB and stored in Apache Spark DataFrame. The next subsections explain the main pre-processing steps.

#### 3.3.1. Irrelevant Characters Removal

We removed Arabic diacritics, punctuation marks. English letters and numbers. For Arabic diacritics, we created a list of all the three forms of diacritics suggested by Diab et al. [81]. The first form is vowel diacritics. It refers to the three main short vowels, named in Arabic as Fatha (ـَ), Damma (ـُ) and Kasra (ـِ) as well as the Sukun diacritic (ـْ), which indicates the absence of any vowel. The second form is nunation diacritics, which are named in Arabic as Fathatan (ـً), Dammatan (ـٌ) and Kasratan (ـٍ). They represent the doubled version of the short vowels. The third form is called Shadda (germination) and refers to the consonant-doubling diacritical (ـّ). It also can be merged with diacritics from the two previous types and result in a new diacritic such as (ـُّ), (ـٌّ). Therefore, the total number of Arabic diacritics is thirteen diacritical marks. All of them will be removed from the text.

Furthermore, we created a list of all the punctuation marks such as commas, period, colons, both Arabic and English semi-colons, and question marks, in addition to the different types of brackets, slashes and mathematical symbols as well as the other signs such as $, %, &, and @. For the hashtags, we strip only the hash (#) and underscore (_) symbols and keep the keywords because it may contain useful information such as the place or event name.
**Algorithm 1** Pre-Processing.**Input:** tweets;  [D]; [P];  [SW] **Output:** Clean, normalized and stemmed tokens**1** $spark  ← createSparkSession()**2** tweets_DF ← spark.read(tweets)**3** **ForEach** tweet in tweets_DF[’text’]  **do**
 //  Remove Irrelevant Characters 
**4**  clean_tweets ← ""**5**  **For** char in tweet **do****6**   **If** char not in [D] AND char not in [P] AND not char.isdigit() AND not char.isEnglishChar() **then****7**    clean_tweets.append(char )**8**  **end**
 // Tokenize
**9**  tokens ← [ ]**10**  tokens ← clean_tweets.split()
 // Normalize
**11**  normalized_tokens ← [ ]**12**  **For** token in tokens **do****13**  **For** alif in [’أ’, ’إ’, ’آ’] **do****14**   **If** alif in token **then****15**    token ← token.replace(alif, ’ا’)**16**  **end****17**  **If** token.endswith(’ ي’) or token.endswith(’ئ’) **then****18**   token ← token.replaceLastCharWith(’ى’)**19**  **If** token.endswith(’ ة ’) **then****20**   token ← token.replaceLastCharWith(’ه’}
 // Remove Stop Words 
**21**  **If** token not in [SW] **then****22**    normalized_tokens.append( token)**23**  **end**
//   Stemmer
**24**  stemm_tokens ← stemmer(normalized_tokens)**25** **End**

#### 3.3.2. Tokenizer and Normalizer

To divide the text into a list of words (tokens), we used a split() method in python, which returns a list of substrings after breaking the giving string by a specified separator, in our case the separator is any white space in the text. After that, the tokens are passed to the normalizer to replace letters that have different forms into the basic shape. The letter (ا) pronounced Alif had three forms (أ, إ, آ). It will be normalized to bare Alif (ا). Besides, the letter (ي) pronounced Yaa will be normalized to dotless Yaa (ى). In addition, the letter (ـة) pronounced Taa marbutah will be normalized to (ـه).

#### 3.3.3. Stop-Words Removal

The Natural Language Toolkit (NLTK) [82] provided a stop-words list for Arabic Language. However, it was designed for the formal Modern Standard Arabic. Therefore, we modified the list and added new stop-words that usually used in dialectical Arabic such as “ليش”, “اللي” and others. Subsequently, we considered common grammar mistakes. For instance, the preposition “متى” might be written “متا” and “لكن” might be written “لاكن”. Besides, we added the common words that are used in Du’aa (prayer) such as “يارب”, “اللهم”, “الله” because they are frequently used and keeping them is not necessary, in our particular case. Before using the final generated stop-words list, we normalized them because they will be stripped from normalized tweets.

#### 3.3.4. Stemmer

The existing Arabic stemmer tools can be categorized into two types, which are root-based stemmer and light stemmer. The first type extracts the root of the words while the light stemmers strip affixes (prefixes and suffixes). However, root-based stemmers are a heavy stemmer and known to have some weaknesses such as increasing word ambiguity. Thus, in this work, we decided to use a light stemmer. However, most of the existing Arabic light stemmers can lead to removing important parts of the word which results in a few letters with no meaning. Therefore, we used the developed Arabic Light Stemmer, Iktishaf Stemmer [6]. It is designed to strip affix based on the length of the word to reduce the chance of stripping important letters which could lead to change the meaning or losing important words particularly the words that are related to transportation. Algorithm 2 shows the algorithm of the proposed stemmer. It gets the normalized tokens, the lists of prefixes [P], and suffixes [S] as input. For prefix, we divide them into three lists, each list contains specific prefixes that do not usually come together in one word. P1 includes prefixes such as “ك”, “و”, “ف”, “ب” whereas P2 contains “ما”, “يا” and P3 includes prefixes such as “ال”, “لل”, “وال”, “فال”, “كال”, “هال”. Both P1 and P2 will be removed only if the token length is greater than 5 to decrease the chance of making mistakes and stripping important letters that are part of the token. For instance, “بريده” city starts with “ب” but the length is greater than 5 so it will not be affected by stemmer. On the other side, any prefixes in P3 will be stripped if the length of the word is greater than 4. Moreover, we take into consideration that the word may contain more than one prefix so the first list of prefixes contains the prefixes that come at the beginning of the word. For instance, the word “ بمايناسب” contains two prefixes: ‘ب’, which is in P1 and ‘ما’, which is in P2 so ‘ما’ will be stripped after ‘ب’.

Similarly, for suffix, we have three lists. S1 contains suffixes such as, “ها”, “كم”, “كن”, “هم”, “هن”, “نا”, “نى”, “ما”, “تن” while S2 contains “ين”, “ون”, “ان”, “وا”. To clarify, we give an example of the verb “يعرف”. It can be ended with any of the following suffixes: كم, ون, هن, نا, ها, نى, هم. It may also contain two suffixes like in “يعرفونهم”, which contains “ون” and “هم” So, “هم” will be removed first because it is in S1 and then “ون”will be removed in the next step.

After removing any suffix from the previous lists, we check the last letter in the word to see if it becomes end with ‘ت’ to replace it with ‘ه’. For example, the word “سيارتهم” (their car) will become “سيارت” after removing “هم” but the correct spelling is “سياره” so we need to replace ‘ت’ with ‘ه’.

Subsequently, the stemmer removes suffix in S3, which are ‘ه’, ‘ى’ only if the length of the word is greater than five and thus, we reduce the chance of stripping them if they are part of the word like the ‘ه’ in the previous example “سياره”. It will not be removed because the word consists of 5 letters. After that, we check if the new stemmed token ends with ‘ت’ to replace it with ‘ه’. For example, the words “سيارتى” (my car) or “سيارته” (his car) will become after stemming “سيارت”, so we need to replace the last letter to make it “سياره”. The final suffix is ‘ات’ and it will be replaced with ‘ه’. For instance, “سيارات” (cars) will become “سياره”. Finally, we check the length of the word after striping suffixes and prefixes and keep only words that have at least two characters.
**Algorithm 2** Stemmer.**Input:** normalized_tokens; [P1]; [P2]; [P3]; [S1]; [S2]; [S3] **Output:** Stemmed tokens**1** stemm_tokens ← [ ]**2** **ForEach** nToken **in** normalized_tokens **do**
// Remove Prefix
**3**  **If** nToken.length() > 4 **then****4**   **If** nToken.length() > 5 and nToken.startWithany([P1]) **then****5**    nToken ← nToken.removeStartWith([P1])**6**   **If** nToken.length() > 5 and nToken.startWithany([P2]) **then****7**    nToken ← nToken.removeStartWith([P2])**8**   **If** nToken.startWithany([P3]) **then****9**    nToken ← nToken.removeStartWith([P3])
\\ Remove Suffix
**10**  **If** nToken.length() > 5 **then****11**   **If** nToken.endWithany([S1]) **then****12**    nToken ← nToken.removeEndsWith([S1])**13**    **If** nToken.endWith(’ا ’) **then****14**     nToken ← nToken.removeEndWith(’ ا ’ )**15**    **If** nToken.endWith(’ ت ’) **then****16**     nToken← nToken.replaceWith(’ ه ’ )**17**   **If** nToken.endWithany([S2]) **then****18**    nToken ← nToken.removeEndWith([S2])**19**    **If** nToken.endWith(’ ا ’) **then****20**     nToken ← nToken.removeEndWith(’ ا ’ )**21**    **If** nToken.endWith(’ت ’) **then****22**     nToken ← nToken.replaceWith( ’ ه ’ )**23**   **If** nToken.endWithany([S3]) **then****24**    nToken ← nToken.removeEndWith([S3])**25**   **If** nToken.endWithany(’ت ا ’) **then****26**    nToken← nToken.replaceWith(’ ه ’ )**27**    **If** nToken.endWith( ’ ت ’) **then****28**     nToken ← nToken.replaceWith(’ ه ’ )**29**  **If** nToken.length() >1 **then****30**   stemm_tokens.append(nToken)**31** **End**

### 3.4. Tweets Labeling Component (TLC)

To generate a training set for the classifiers, we need labeled tweets. Since we have a very large dataset of around 33.5 million tweets, manual labeling will be very expensive and time-consuming process. We manually labeled approximately twenty thousand tweets of the total 33.5 million tweets and then we combined them with automatically labeled tweets using the automatic labeling approach. The manually labeled tweets also help us to generate dictionaries for automatic labeling since it is a lexical based approach. The following subsections explain the proposed approach for labeling tweets about event classifiers and tweets filtering classifier. Even though we detect events after filtering tweets, we will start with labeling events because the output will be used later on to label the tweets into relevant and irrelevant.

#### 3.4.1. Automatic Labeling for Events Tweets

##### Creating Dictionaries

For each event type, we automatically generated a dictionary that contains the top frequent terms using the manual labeled tweets. We manually updated each dictionary to include the missing terms related to each event type in addition to add synonyms. Both manually and automatically added terms in the dictionaries are passed to the stemmer since the search for matching terms will be applied to the tweet after pre-processing. We used Iktishaf light stammer (see Section 3.3.4).

The dictionaries contain a group of terms but we cannot use them directly to search for matching tweets because the degree of relevance to the event type is not equal for all the terms. Therefore, for each event type, we created an N number of terms list. Each list is considered as a level. So, we have N number of levels (L_1_, L_2_, ..., L_n_). Each term T in the event dictionary is assigned to a level based on the degree of the importance of this term to the event. Thus, the terms that are highly related to the event and almost exist in each report about this event are assigned to the first level (L_1_) while the last level contains terms that are least related to the event.

Furthermore, we gave each list a weight W based on the level it belongs to, which means we have N weights (W_1_, W_2_, …, W_n_). W_n_ is the highest weight so it is assigned to L1 which contains the most important terms. In this work, we used 4 levels of terms. To clarify, for Accident event, Level 1 includes terms such as Accident (حادث) and crash (صدم), Level 2 contains terms such as car (سياره), driver (سائق) and, road (طريق), Level 3 include Ambulance (اسعاف) and death(وفاه) while the last level (Level 4) contains the less important/relevant terms such as cause (يسبب).

Algorithm 3 shows the automatic labeling algorithm. It receives the pre-processed tweets (tweets_P), term dictionaries (terms_D), and event types (event_T) as input and provides the labeled tweets as an output. Apache Spark is used and the pre-processed tweets are stored in Spark Dataframe (tweets_DF). For each token in the tweet, it searched for the matched term in the terms dictionary. The “Find Matching Terms” section explains the process of searching for the matching terms. After that, weight is calculated for each labeled tweet. The section “Weight Calculation” clarifies the process of weight calculation. The last step is sorting and filtering the labeled tweets based on the calculated weight, see section “Sort and Filter Automatic Labeled Tweets” for further details.

##### Find Matching Terms

For each tweet, we applied the pre-processing steps explained in Section 3.3 to remove irrelevant characters, divide the text into tokens, normalize the tokens, remove the stop words, and apply stemmer. The output is N number of clean normalized and stemmed tokens (K1, K2, …, Kn). Moreover, we iterated over each tweet and for each token K, we searched for the match terms in the term levels (L_1_, L_2_, …, L_n_). The output is a list of existing terms in each level as shown below where Tx is the matching term:

(1) $$Lx_{1\;}=\left[Tx_{1\;},\;Tx_{2},\;\ldots ,\;Tx_{n\;}\right],\ldots ,Lx_{n\;}=\left[Tx_{1\;},\;Tx_{2},\;\ldots ,\;Tx_{n\;}\right]$$

**Algorithm 3** Automatic Labeling for Events.**Input:** tweets_P; terms_D; event_T **Output:** Labeled tweets**1** spark ← createSparkSession()**2** tweets_DF ← spark.read(tweets_P)**3** n ← get_levels_num(terms_D)**4** **ForEach** tweet **in** tweets_DF **do****5**  tokens ← get_tokens(tweet)**6**   **forEach** event **in** event_T **do****7**     term_levels ← terms_D[event]**8**    **forEach** token **in** tokens **do****9**      l ← 1**10**      stopSearch ← False**11**      **while** l < n **do****12**       **If** !stopSearch **then****13**        **ForEach** term **in** term_levels[l] **do****14**         **If** term.isEqual(token) **then****15**           matchedTerms ← add(term,l)**16**           stopSearch ← True**17**           break**18**      **end****19**     l ← +1**20**    **end****21**   **end****22**   tweet_weight[event] ← calculate_weight(matchedTerms)**23**  **end****24**  tweets_DF[’weight’] ← tweet_weight**25** **end****26** tweets_label_DF ← sort_and_filter(tweets_DF[’weight’] )

##### Weight Calculation

We filtered the tweets to keep only the tweets contain at least one term from the high-level (L1), except for roadwork/damage event type, we keep tweets that contain at least one term from level (L1) and at least one term from level (L2). For roadwork/damage event, L1 includes terms such as maintenance (صيانه), development (تطوير) while L2 include terms such as road (طريق), street (شارع). So at least on terms from each level should be found to assign a label to a tweet. After that, the weight is calculated using the following equation:

(2) $$W_{E}=\left\{\left(size\left(Lx_{1}\right)\times W_{n}\right)+\left(size\left(Lx_{2}\right)\times W_{n-1}\right)+\ldots +\left(size\left(Lx_{n}\right)\times W_{1}\right)\right\}$$

where *W_E_* is the total weight for the event *E*, *Lx* is the list of matching terms and *W* is the weight assigned for this level. Since we have 4 levels, the highest weight is 4, so, each of the term Tx that was found in Level *Lx*_1_ has a weight equal to 4.

##### Sort and Filter Automatic Labeled Tweets

We used the weight to sort the labeled tweets and then we filtered them. Furthermore, we specified a threshold to discard labeled tweet that has low weight and kept tweets that have high weight because they are most likely related to the event. The same process is repeated for each event type. We take into consideration during this process that the tweet can have multiple labels.

##### Testing and Evaluation

The testing and evaluation of the proposed automatic labeling tool are performed in two stages. The first stage is in the beginning in order to update and modify the list of terms in each level in the dictionary such as moving terms from level to another level or adding new terms. After each initial labeling iteration, we extracted the top vocabularies to search for the new important terms that are not yet included in the dictionary of that event. For instance, for the first iteration, most of the weather event is about rains so the automatic generated dictionary contains terms related to rains. So, this stage is important to insert missing terms and then we manually update the dictionary to add their synonyms if they exist. The second stage is applied to randomly selected tweets to make sure that the tweets are labeled correctly. The main goal is reducing the number of false positive (labeled as an event but it is not) more than the number of false negatives (event but labeled as not related) to reduce the chance of making mistakes and including none event tweet in the training set of events classifiers. Besides, missing a few events tweets will not have a major effect on the size of the training set.

#### 3.4.2. Automatic Labeling for Irrelevant Tweets

Before detecting the event, we need to train a classifier to classify the tweets into relevant and irrelevant to traffic. The training set contains positive tweets (related to traffic) and negative tweets (not related). Even though we train the filtering classifiers before the events classifiers, we generate the training set for event before filtering classifiers. The output from the automatic event labeling process is used for a positive class for tweet filtering. For negative class, we applied another automatic filtering approach by searching for the tweets that do not contain any terms related to traffic and transportation. We searched in the tweets collected by geo-filtering and we excluded tweets posted by any account related to traffic.

### 3.5. Feature Extractor Component (FEC)

We used CountVectorizer and IDFModel algorithms provided in the Spark ML package to generate the feature vectors and rescale them. IDFModel applied TF-IDF (Term Frequency-Inverse Document Frequency), which reflects the importance of a token to a document (tweet) in a corpus. The TF-IDF is the product of TF and IDF where TF (t, d) is the frequency of the appearance of token t in document d while the IDF is calculated using Equation (3). A detailed explanation was given in our earlier paper [6]:

(3) $$\mathrm{IDF}\left(t,\;D\right)=log\frac{\left|D\right|+1}{\mathrm{DF}\left(t,D\right)+1}$$

### 3.6. Tweets Filtering Component (TFC)

#### 3.6.1. Model Training

To filter tweets into related to road traffic and not related, we built a classifier using ML supervised classification algorithms. After labeling the tweets using both automatic and manual labeling approach, we used the Spark ML library to build and train the models. We built three models using SVM, naïve Bayes and logistic regression algorithms. We have an imbalanced dataset because in our work, the number of samples for the negative class (not related to traffic) is much higher than the positive class (traffic-related). This will lead to misleading evaluation results, especially for accuracy. To address this issue, we applied a random under-sampling approach to randomly remove some tweets from the negative class.

#### 3.6.2. Hyperparameter Tuning

After data processing and feature extraction, we need to tune the parameters to obtain the best performance model. Grid search is one of the well-known ways to search for the best tuning parameter values. To do that, we need to specify a set of candidate tuning-parameter values and then evaluate them. Cross-validation can help to generate samples from the training set to evaluate each distinct parameter value combination and see how they perform. After that, we can get the best tuning parameter combination and use them with the entire training set to train the final model.

Spark ML supports model selection using tools such as CrossValidator to select a relatively reasonable parameter setting from a grid of parameters. We used 5-fold cross-validation, so CrossValidator will generate five (training, testing) dataset pairs. Then, the average evaluation metric for the five models will be computed and the best parameter will be founded. In the future, we plan to improve our method and use 10-fold validation.

#### 3.6.3. Classification Model Evaluation

We compare the performance using the common evaluation metrics, which are accuracy, recall, precision and f-score. The model that achieves higher results is selected for the final classification of tweets. Since we are using binary classification to classify into relevant (class 1) and irrelevant (class 0), tuning the prediction threshold is very important. The default threshold is 0.5 and it can be any value in the range [0, 1]. If the estimated probability of class label 1 is greater than the specified threshold, the prediction result will be 1, otherwise, it will be class 0. Thus, specifying a high threshold value will encourage the model to predict 0 more often and vice versa. In our case, we need to minimize the chance of making mistakes and predicting 0 (irrelevant) as 1 (relevant) so we set the threshold to 0.8.

### 3.7. Events Detection Component (EDC)

We focus in this work on detecting the following event types: Fire, Weather, Social, Traffic Condition, Roadwork/Road Damage, and Accident. The tweets are labeling using the labeling method explained in Section 3.4. The classes are not mutually exclusive where the tweet can be about multi-events at the same time. For instance, the tweet might explain the accident that occurs due to bad weather. Hence, two labels will be assigned to this tweet, which are accident and weather. To address the issue, we used a binary classification. We trained a model for each event type. For each model, we need positive and negative samples. Assume we have event type T, the tweets that are labeled as T considered as positive samples while all the remaining tweets that belong to the other events types are considered negative samples. Moreover, tweet that has more than one label such as accident and weather will be included in the positive class in the training set of accident as well as weather during training both classifiers. However, as the number of tweets on the negative class is very large compared to the positive because it includes all the tweets about the other events types, we have an imbalance dataset problem. To address this problem, we followed the same approach explained in Section 3.6.1 by applying a random under-sampling approach.

### 3.8. Spatio-Temporal Extractor Component (STEC)

The location is the foremost matter of interest in transportation analysis and event detection domain. Thus, we applied different techniques for location extraction from the Tweet object.

#### 3.8.1. Text, Hashtag and Username

The main approach is extracting location details which are mentioned within the post. It might be explicitly mentioned in the tweet’s message or it might exist as part of the hashtags or accounts name especially if the tweets are posted by a specialized account that posts about the events and traffic condition in the cities. We created a list of cities name in Saudi Arabia to search for cities name in the tweet message. We pass the Arabic names list to the stemmer before using them to extract the place name from text because we extract them from pre-processed text. In addition, we searched for the cities name in English to extract them from accounts or hashtags using a predefined list of cities’ names in English. We also created a list of specialized accounts that post about traffic in Saudi Arabia cities and does not include a city name. After that, we use this list to find the city name based on the username.

#### 3.8.2. Tweets Geo Attributes

One of the approaches is obtaining coordinates in geotagged tweets by getting latitude and longitude from ‘coordinate’ or ‘place’ objects. The ‘place’ child object consists of several attributes including ‘place_type’, ‘pl ce_name’, ‘country_code’. The place type is either city or point of interest (poi). However, a small fraction of tweets are geotagged because most of the users used to disable location services in their smartphones for privacy reasons.

#### 3.8.3. User Profile

The location information is also extracted from user profiles where they usually manually write the country and city name. We have to consider that this information might be written in Arabic or English and they use different spelling. For instance, Makkah can be written as Makkah or Mecca. The text is tokenized and then passed to the stemmer before searching for the city name using the created dictionaries.

We cannot rely on the geo attributes alone because geo coordinates information might not have been provided especially for users who disable location services in their smartphones where the value will be ‘null’ in this case, as shown in the JSON example in Section 3.2. Similarly, we cannot rely on profile information alone because users do not always fill in these fields with accurate information. In addition, they might travel to another city/country so the profile information, does not reflect the current location. Besides, both approaches do not necessarily represent the place of the event because users might post about events that occur in other cities.

In this work, we considered the text as the main source of location information because it is more accurate than the other attributes besides, we need to find the location where the events occur not where they were posted. If the information does not exist, we extract them from coordinates or place attributes. The last option is to find location from the profile because it is less accurate than the other since users specify their information in the profile manually and they do not usually update them whenever they travel to another city. For visualization, the geospatial coordinates of the detected locations are extracted to enable plotting them on the map.

### 3.9. Reporting and Visualization Component (RVC)

This component supports plotting the output of the spatio-temporal information extraction component and event detection components to show the detected events and their location and time of occurrence. Also, it supports finding peak events based on configurable parameters as well as visualize the results. Algorithm 4 shows the peak events reporting algorithm. It enables searching for hourly, daily and monthly peak events when the tweets intensity exceeds a specific threshold value.

Moreover, this component supports visualizing the results of the model evaluation (see Section 3.6.3) for both tweet filtering and event detection components to illustrate which algorithm achieved better results.
**Algorithm 4** Peak Events Reporting.**Input:** event_Tweets; event_Types; threshold; duration **Output**: Peak events list **1** **for** event **in** event_Types **do****2**  **for** d in duration **do**
**//** Duration can be Hours, Days or Months
**3**    peakEvent← [[[]**4**    **for** tweet in event_Tweets[event] **do****5**     **if** tweet.intensity() >threshold **then****6**      peakEvent.append( tweet )**7**    **end****8**  **end****9** **End**

### 3.10. External and Internal Validation Component (EIVC)

To validate Iktishaf+ tool and its ability to detect events and their spatial and temporal nature. We searched against various sources on the web including news media. Then, we compared the information extracted by our tool with the information in the web sources. However, news media do not report all the existing events and even if they report them, they might not mention the time of occurrence. In this case, we searched in the tweets we have, related to the event, to find the validation information we need. We consider this process as an internal validation because it is based on the collected tweets. To find time information, we go back to the earliest tweet we have about the event and if the time is not mentioned explicitly in the tweet text, we refer to the time of posting the tweet as the starting time of the event. The process of searching in the external sources was done manually, but we plan to automate it in the future.

## 4. Analysis and Results

### 4.1. Detected Events

#### 4.1.1. Validation of Detected Events

To validate the ability of iktishaf+ and verify if a detected event really happened on the same detected date and location**,** we searched against external validation sources (news media) or an internal source (Twitter) (see Section 3.10). Table 1 shows a comparison between information extracted by Iktishaf+ and information from external/internal sources. We cannot discuss all the detected events, due to the limited number of pages allowed and the large period we are covering in this work (September 2018–October 2019). So, we selected samples of different event types occurred in different time and location. Column 1 shows the event types. Column 2 lists the location (city name) where the event occurs as we found from searching in various external sources as well as the location extracted by our tool. Column 3 gives the date when the events occur. Column 4 gives the time of occurrence to assess the ability of our tool to detect the time. We compared the starting time mentioned in the web sources with the peak time showing by Iktishaf+. As explained in Section 3.10 the time information may not be mentioned in the news reports. So, in this case, we searched in the collected tweet and get the earliest tweet about this event. Then, we extracted the time from the timestamp attached to the tweet.

Moreover, we drew charts to display the time extracted by Iktishaf+ for each event in the table. Further, the locations of each event are overlayed on top of the Saudi Arabia map.

**Figure 4:**
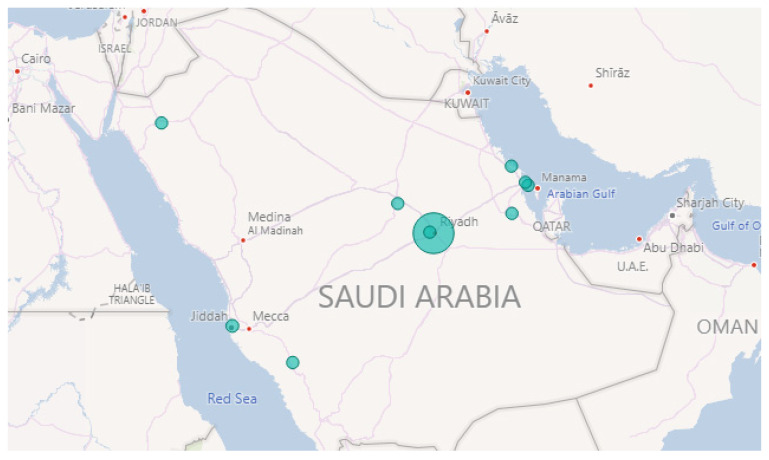
Fire Event on 1 October 2018.

**Figure 5:**
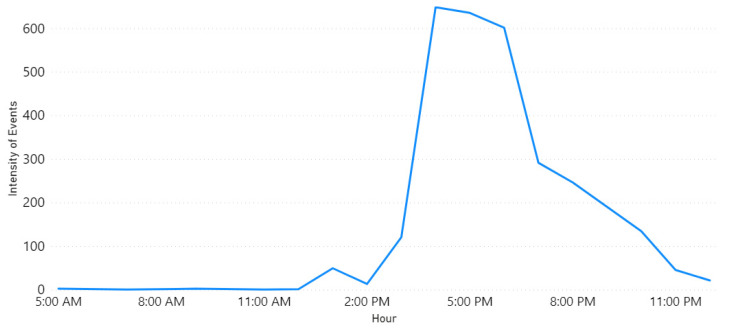
Intensity of Detected Fire Event in Riyadh (1 October 2018).

Row 1 shows the “Fire” event on 1 October 2018. Figure 4 illustrates the locations. Note the largest circle in Riyadh city, this matches the information found in the newspaper, where they reported about a huge fire that broke out at a power plant in Riyadh [83]. They also mentioned that the Saudi Civil Defense received notification about the fire at 3 p.m. Figure 5 shows the time extracted by our tool. It can be seen that the intensity started raising at 3 p.m. and the highest peak was at 4 p.m.

**Figure 6:**
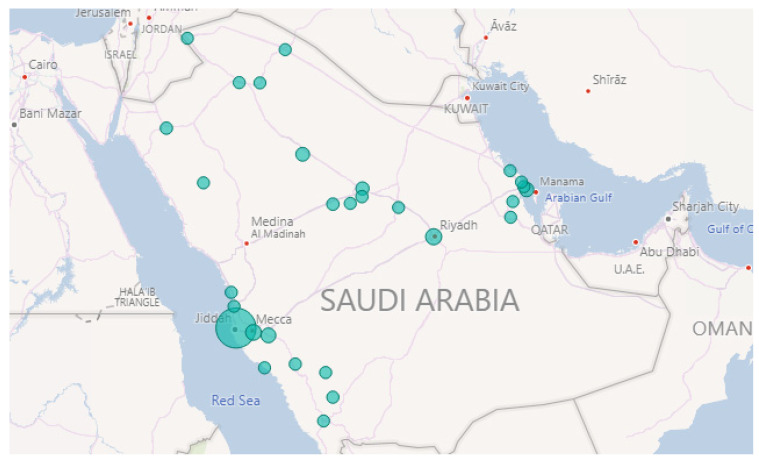
Fire Event on 29 September 2019.

**Figure 7:**
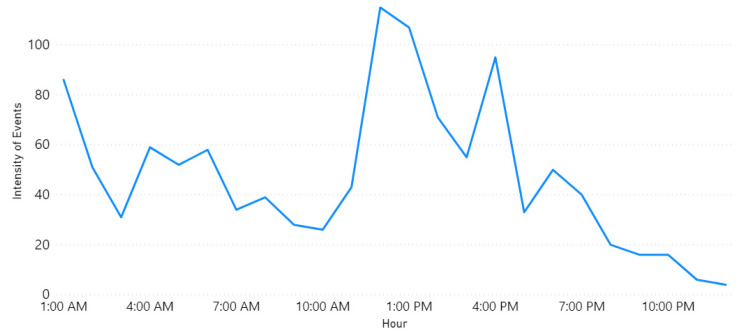
Intensity of Detected Fire Event in Jeddah (29 September 2019).

Row 2 validates another “Fire” event. As we found in the web source [84], it was a massive fire that ripped through the main station of the Haramain high-speed railway in Jeddah city on 29 September 2019. It started at 12:35 p.m. according to the Haramain high-speed railways’ Twitter account. Further, it burned for hours before it was brought under control. Figure 6 and Figure 7 show the location and time information extracted by Iktishaf+. Note the largest circle in Jeddah city as well as the peak time around noon as shown in Figure 7.

**Figure 8:**
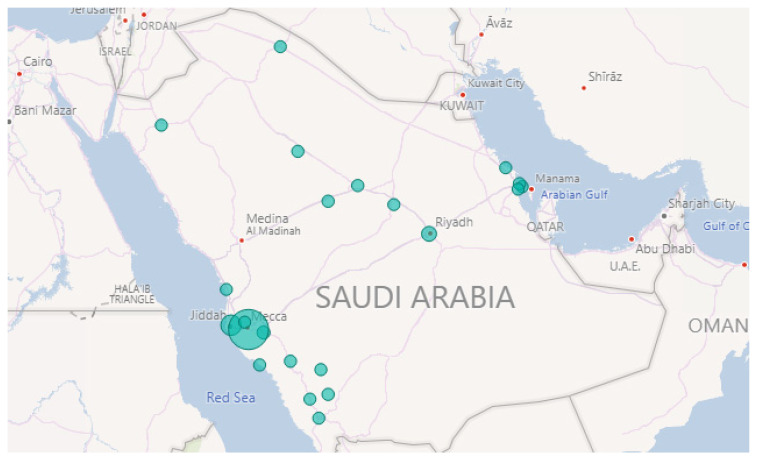
Weather Event on 23 November 2018.

**Figure 9:**
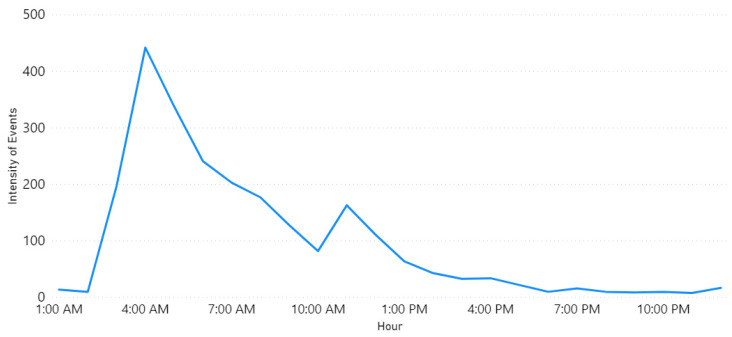
Intensity of Detected Weather Event in Makkah (23 November 2018).

Furthermore, Row 3 validates the “Weather” event on 23 November 2018. This was due to the rains in Makkah and Jeddah cities as reported in the newspaper [85]. Figure 8 plots the locations of weather events on that date. Note the largest circles are in Jeddah and Makkah cities. The news article we found was posted around 5:42 a.m. and they mentioned that the rains started at dawn. This validates the time extracted by our tool. Figure 9 shows a peak at 4 a.m. As we know, Twitter users almost post about events like rain immediately once they happen and most likely earlier than newspapers.

**Figure 10:**
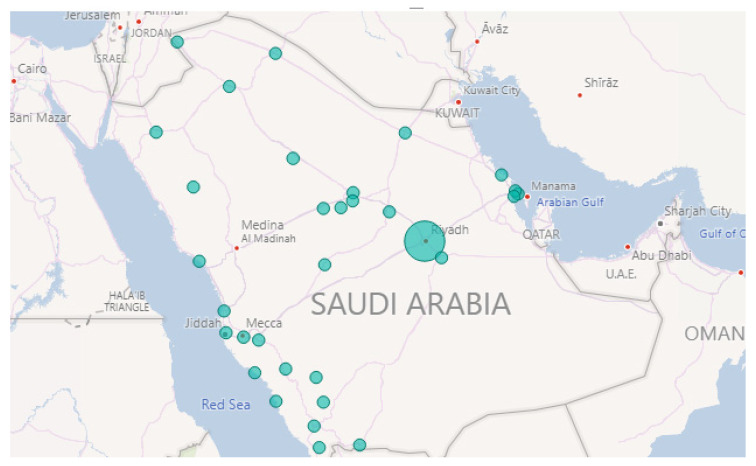
Accident Event on 8 October 2018.

**Figure 11:**
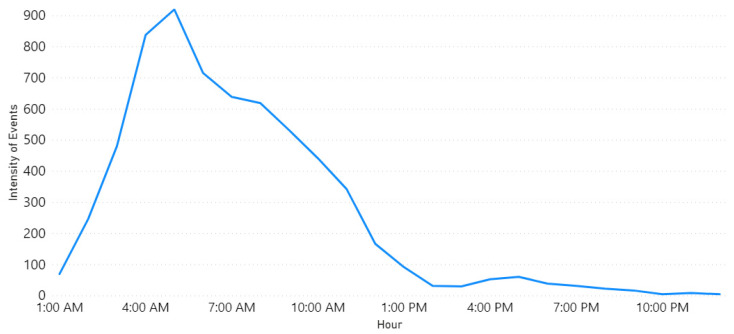
Intensity of Detected Accident Event in Riyadh (8 October 2018).

Finally, Row 4 illustrates the “Accident” event on 8 October 2018. Note the largest circle in Riyadh shown in Figure 10. The time of occurrence is not available on the newspaper website so in this case, we went back to the tweets and searched for the earliest tweet that mentioned information about the same accident we wanted to validate. Then, we extracted the time from the timestamp (created_at attribute) included with the tweet object and assumed that it was the time of occurrence (see Section 3.10). This is the English translation for the earliest tweet we found about this event “Congestion in every street and accident in the Alwashm bridge, stations crowded, crowded everywhere in Riyadh #Riyadh_now”. The time attached to this tweet is “Mon Oct. 08 04:48:17 +0000 2018” and the first peak time detected by Iktishaf+ as shown in Figure 11 is at 5 a.m. Therefore, it can be seen from the discussed results above that the information from external or internal validation sources matches the information detected by Iktishaf+, which proves the ability of our tool to automatically detect events and their location and time without prior knowledge.

#### 4.1.2. Spatial Analysis

**Figure 12:**
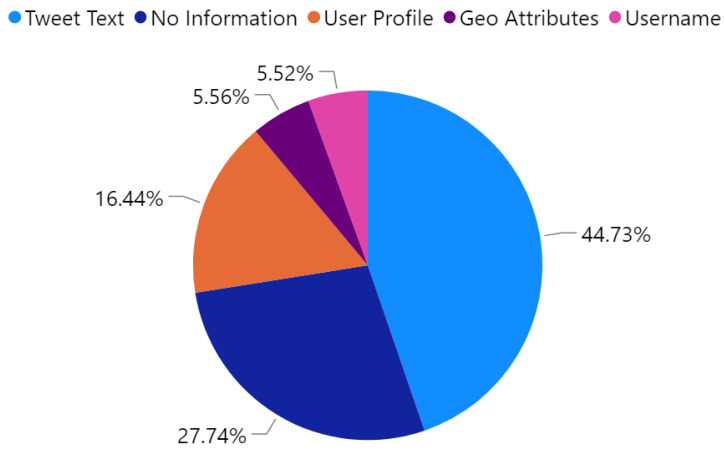
Number of Tweets Using Different Location Extraction Approaches.

Figure 12 depicts the percentage of the extracted location information using different approaches explained in Section 3.8. As shown in the pie chart, 44% of the information is extracted from tweet text while 16% are extracted using the information in the user’s profile. However, 27% of tweets about events did not include any information about the location where it occurs. Besides, only 5% are extracted from geo attributes. This could be because few tweets are geo-tagged because users usually turn off the location service in their smartphones. Also, we only look into the geo attributes if the location does not mention in the text because we mainly focus on where the event occurs not where it has been posted.

**Figure 13:**
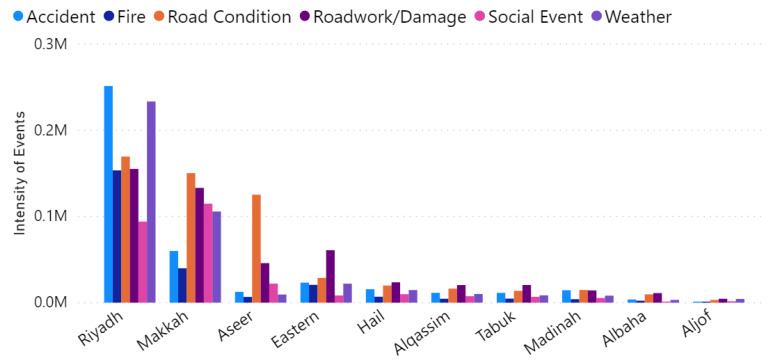
The Number of Detected Events in Different Provinces.

After inferring cities’ name from the tweets, we group them by province. Figure 13 gives the number of tweets for each event type in the large provinces in Saudi Arabia. It shows the aggregated number of tweets for the whole period (from September 2018 to October 2019). It can be seen that the number of events detected in Riyadh is higher than the events in other provinces. This could be because Riyadh is the capital and one of the largest cities. Besides, based on the latest report published by INREX [86], Riyadh is the most congested city in Saudi Arabia, which may explain the results we got.

#### 4.1.3. Spatio-Temporal Analysis

**Figure 14:**
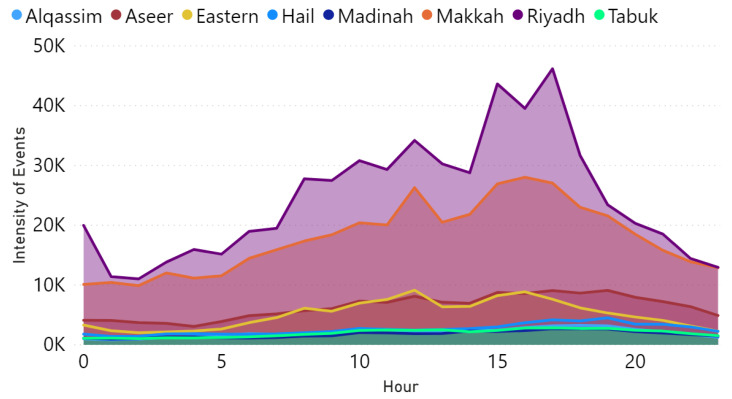
Hourly Distribution of Tweets Divided by Provinces (Aggregated).

Figure 14 shows the hourly distribution for the aggregated number of tweets for the whole period. We plot only the provinces that show the high number of events to eliminates having too much data in the chart since we have 13 provinces in Saudi Arabia. As shown in this figure, the number of tweets starts raising in the morning and becomes very high by the time of coming back from school and work, which usually between 12 p.m. and 5 p.m. Further, the number goes down after 8 pm, which is expected because usually the traffic flow and activities during day-time are higher than the night-time because of work and schools.

### 4.2. Evaluation: Tweet Filtering Classifiers

**Figure 15:**
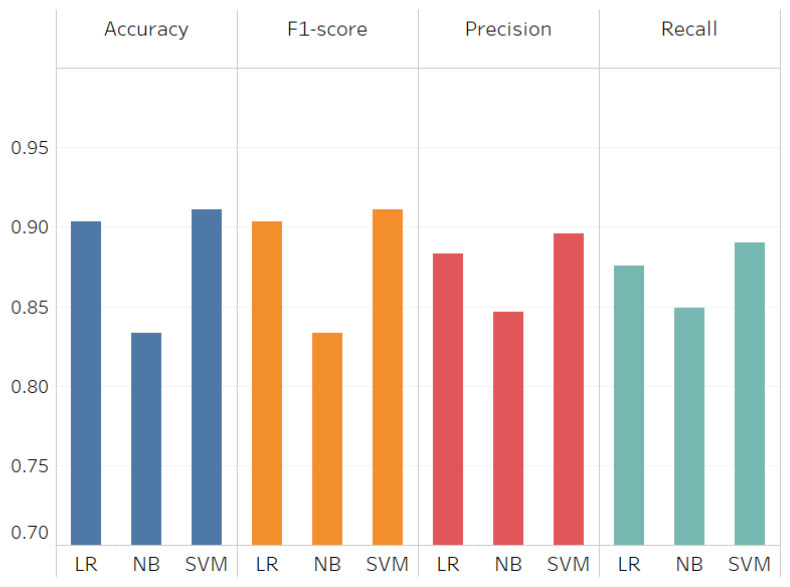
Numerical Evaluation (Tweets Filtering).

To evaluate the trained model for the Tweets Filtering Component (TFC), we used the common statistical metrics, accuracy, precision, recall, f-score (see Section 3.6.3). Most of the tweets we have are irrelevant to traffic, so we have an imbalanced dataset. So, to eliminate the effect on the evaluation results, we have two options either oversampling the majority class that represents the irrelevant tweets or under-sampling the minority class that represents the traffic tweets. In our particular case, it is better to have a large number of samples for both classes. Therefore, we decided to apply oversampling on positive (traffic-related) class. We simply duplicate the number of the tweet in the positive class (see Section 3.6.3). Figure 15 shows that SVM achieved higher results compared to the other algorithms. It achieved 91% for both accuracy and f1-score, 90% for precision and 89% for recall. The difference between the results achieved by SVM and LR is approximately 1%. However, we selected SVM where it performed better.

### 4.3. Evaluation: Event Classifiers

We numerically evaluated the built binary classifiers in the Event Detection Component (EDC). For each event type, we trained three models using NB, SVM, and LR algorithms and then we selected the algorithm that achieved higher results in most evaluation metrics (accuracy, precision, recall and f1-score). We have used the four performance metrics as discussed before in the previous section (also see Section 3.6.3).

**Figure 16:**
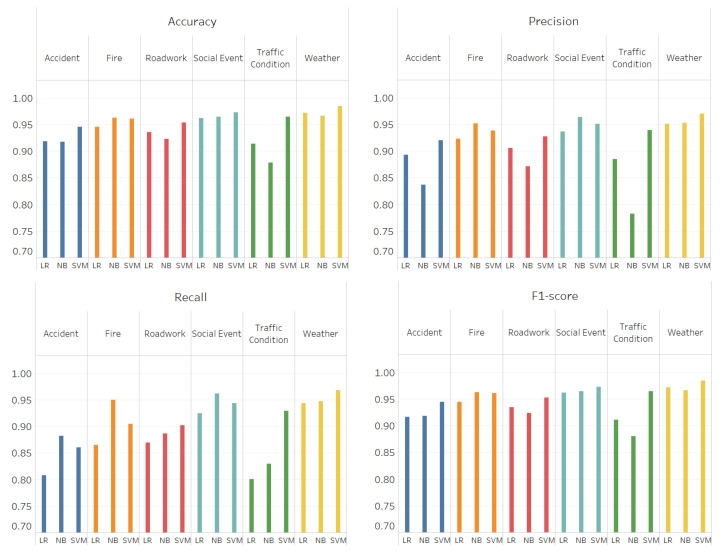
Numerical Evaluation (Events Classification).

Figure 16 illustrates the evaluation results for the four performance metrics (left to right: accuracy, precision, recall, and F-score) in four separate figures. The results show that SVM performs better than other algorithms for Weather, Roadwork and Traffic condition events while NB performed the best for Fire event. Besides, for accident events, SVM achieved higher results for accuracy, precision and f1-score while NB performed slightly better for the recall where SVM achieves 86% whereas NB achieves 88%. However, since the SVM performed better for most metrics, we selected SVM for accident event. For Social event, NB achieved higher recall and precision while SVM performed better for accuracy and f1-score. However, we selected NB for Social events since the accuracy and f1-score achieved by SVM are approximately 1% higher than NB. To summarize, SVM has been used for all the event types except Fire and Social where we used NB. Moreover, the highest results we got in all metrics were achieved by SVM for Weather event where it achieved 98% for both accuracy and f1-score and 97% for recall and precision. We assume that the reason is we have a larger number of tweets for the training set of weather event since it occurs more often and a lot of users post about it compared with other event types such as accident and roadwork/damage.

## 5. Conclusions and Outlook

Digital societies could be characterized by their increasing desire to express themselves and interact with others. This is being realized through digital platforms such as social media that have increasingly become convenient and inexpensive sensors compared to physical sensors in many sectors of smart societies. One such major sector is road transportation, which is the backbone of modern economies and costs globally 1.25 million deaths and 50 million human injuries annually. The cutting-edge on big data-enabled social media analytics for transportation-related studies is limited.

In this paper, we introduced the Iktishaf+ tool that uses big data and distributed machine learning to automatically detect road traffic events from Arabic tweets. Manual labeling is a time-consuming process that makes supervised classification hard to apply to big data. In order to address this problem; we proposed an automatic labeling approach to reduce the effort and time of generating a training set for training supervised classification models. The traditional manual labeling for text is usually achieved by looking for specific terms to decide whether the text is relevant to the topic or not. Hence, our tool was designed to follow the same procedure. We built a dictionary for each event type that contains lists of terms that usually used when posting about the events. The dictionaries were generated automatically using the top vocabularies extracted from the manual labeled tweets. Then, we updated them manually to add synonyms and missing vocabulary. After that, we divided them into levels based on the degree of importance and relevance to the event type. Subsequently, the tool looked up the matched terms and labels the tweets based on that. Finally, the tool calculated weight for each labeled tweet, and only tweets that are highly related to the event are included in the training set.

Furthermore, we developed a location extractor to find the location of the events allowing spatio-temporal information extraction and visualization of the events. Moreover, using a stemmer is necessary for our work not only to minimize feature space for model training but it is also very helpful especially for terms searching during automatic labeling and location extraction. The existing Arabic stemmers are not efficient in our case where they might lead to removing an important letter from the word and then cause losing or changing the meaning of important words. Therefore, we designed a light stemmer that enables affix stripping with fewer changes in the word meaning.

We built and trained models to filter out irrelevant tweets to traffic events. We focused on six events that might affect road traffic which are accident, fire, weather, roadwork/damage, road condition, and social events. Furthermore, we built classifiers to automatically classify tweets into different events. We used three machine learning algorithms, which are SVM, NB, and logistic regression. Then, we selected the algorithm that achieves better results in terms of accuracy, recall, precision, and f-score. Moreover, we applied external validation using online sources such as newspapers. We selected the highest peaks from the detected events and find whether they occurred or not. The results show that our tool is able to automatically detect events and their spatial and temporal nature without prior knowledge.

The ability of the Iktishaf+ tool to use big data distributed computing technologies could save days, months, or years of computing time proportional to the size of the data. Moreover, it enables the scalability and interworking of big data analytics software systems. The utilization possibilities of our tool are many such as detection of transportation-related events for planning and operations, detection of causes of road congestion, understanding public concerns and their reactions to government policies and actions, and many more. An elaboration of these aspects of our work (the novelty, contributions, and utilization) was given in Section 2.4.

We have shown good evidence of the use of automatic labelling, machine learning, and other methods. However, more work is needed to improve the breadth and depth of the work with regard to what can be detected, the diversity of data and machine and deep learning methods, the accuracy of detection in space and time, and the real-time analysis of the tweets.

The real-time operation of the proposed system could depend on a number of factors. Firstly, the definition of the term “real-time” per se depends on the application and the requirements at hand. Some applications may require reactions within sub-second periods while others may tolerate a few minutes or more. Moreover, taking preventive actions also depends on the event and the action being taken. In this particular context, and considering the example of a car accident, the Iktishaf system once trained can detect an accident from tweets instantaneously provided the tweets are available in real-time for the software to process. This can be achieved, for instance, by running the software at the edge or fog layers. The reactive actions, in this case, can mean to inform the police and ambulance services, which can be done in real-time by the software automatically through an automatic emergency call to 911, by sending tweets or other messages to the concerned bodies, or by other emergency strategies available in the area. The messages related to the particular actions in this context can also be propagated using vehicular ad hoc networks (VANETs), dedicated short-range communications (DSRC), etc. A more interesting and lucrative work would be to detect certain events (such as car chases or certain patterns in the traffic that may lead to accidents or certain social events that may cause congestion) before these happen and take actions to prevent the events before they happen. These will require further research and adding additional functionalities to the Iktishaf tool. Our future work will look into these areas.

Digitally and data-driven methods while bringing many benefits to the research and practice have their risks and disadvantages as is the case for anything else. These include issues related to security, privacy, data ownership, lack of standards describing ethical requirements from digital methods and compliance to these standards, the safety of the stakeholders involved in data-driven and digital methods, vulnerabilities of digital platforms, and the digital divide. For a detailed discussion of these issues, see Section 11 in [87], and the references therein. As regards the specific privacy issues of Twitter data, the data we use is openly available. The information about the location of these tweets is also public. However, we have not disclosed any personal information through our analysis. The information we detected and published is of general nature and therefore does not infringe on individual privacy. However, generally speaking, it is possible to detect information from Twitter data that affects individuals’ privacy. Our earlier works [57,88,89] have looked into privacy and we plan to extend this to investigate Twitter data privacy in the future.

Our focus in this work is on Saudi Arabia. The tool hence currently works with tweets only in the Arabic language. The tool can be used in other Arabic language-speaking countries, such as Egypt, Kuwait, Bahrain, and UAE. The system methodology and design of the tool developed in this paper are generic, and therefore the tool can be extended to other countries globally. This will require the adaptation of the tool with additional languages, such as English, Spanish, or Chinese, by additional modules in the pre-processing and clustering modules.

This line of our work deals with the use of Twitter data as a virtual sensor to detect transportation-related events. It is necessary to look also into other sources and methods of sensing in transportation systems, such as inductive loops, floating car data, automatic vehicle locators, virtual loop detectors, cooperative driving, etc. The real vision and potential of smart transportation systems will be realized when different sensing systems will be integrated within the transportation systems as well as with other urban systems. Our other strands of research have looked into different traffic sensing methods such as GPS [51], inductive loops [49], cooperative decision-making for autonomous vehicles [35], and urban travel data from travel cards and other sources [50]. Our future work will look into integrating these sensing methods along with other urban sensing systems, such as healthcare [7].

## Figures and Tables

**Table 1:** Example of Validation.

Event Type	Location		Date		Time	
	Validation Sources	Iktishaf+	Validation Sources	Iktishaf+	Validation Sources	Iktishaf+
Fire	Riyadh		1/10/2018		Started at 3 p.m.	Found peak around 4 p.m.
	Jeddah		29/9/2019		Started at 12:35 p.m.	Found peak around 12 p.m.
Weather	Makkah and Jeddah		23/11/2018		Started at dawn (before 5:42 a.m.)	Found peak around 4 a.m.
Accident	Riyadh		8/10/2018		Started around 4:48 a.m.	Found peak around 5 a.m.

## Data Availability

Data was obtained from Twitter. Restrictions apply to the availability of these data.
